# Medical Protection for School Morals

**Published:** 1894-03-31

**Authors:** 


### The Hospital

An Institutional Journal of

The Medical Sciences and Hospital Administration, with

Vol. XV.—No. 392.] AN EXTRA NURSING SUPPLEMENT. [March 31, 1894.

### Medical Protection for School Morals.

It is our practice from time to time to direct attention to important physiological aspects of public school life. Those boys who are sent from home at ages when they are little more than infants to preparatory schools, and who from that time onwards know no other real life but that of the preparatory or the public school until they are on the near verge of manhood, have many claims upon the consideration of men of science and men of the world as well as upon that of their parents and responsible guardians. There must be something essentially manly in English boyhood, and essentially excellent in English public school life, when so large a proportion of the young of our prosperous classes pass through this trying period of their development and emerge wholesome in body and mind, and imbued with principles and aims worthy of the noblest type of manhood.

But the ordeal is sometimes fiery. A medical contemporary announces that "a member of council of a large school has forwarded an abominable circular, which was addressed to one of his boys, offering to pay ten shillings for every name and address of male persons suffering from any complaints of the generative organs, kidney or bladder diseases, or nervous debility in any form whatsoever—that is, if after this unfortunate creature has been flooded with circulars an order is received." Now there cannot be two opinions about the baseness of persons who follow such a trade as this. But our concern is not with them. There cannot be two opinions about the necessity for putting an absolute stop to all such methods of contaminating and debasing the minds of boys. The thing imperatively demands to be done. The question is, How is it to be done effectually, and who is to do it? Our contemporary appeals to Government. This is tantamount to a confession that it has nothing of a practical nature to suggest. In a matter of this kind an appeal might as well be made to the Grand Llama of Thibet, who, if travellers lie not, passes his life in the secure enswathement of an amplitude of cotton wool. It would be well if the medical profession would understand at once that "Government" in our democratic times suffers almost incurably from two maladies of a very hopeless character; on the one hand it is overwhelmed with an immeasurable plethora of work; on the other it is disabled by an increasing paralysis of governing energy. These are no mere phrases; they are the plain truth. How then can such a Government take the initiative in any kind of new legislation, especially of legislation of a highly specialised kind, demanding special knowledge, very special care and wisdom, and a very high degree indeed of special moral energy and enthusiasm combined with public spirit?

It is a contention often reiterated by us, and yet to be reiterated until practical results are obtained, that the medical profession, in its corporate capacity, is a definite department of the State, is in fact the church or the law of the body. It must learn to act in that capacity. It owes certain obligations to the State by virtue of its special knowledge and practical powers, which it can only fulfil by working as one single, united, effectual organisation. The duty we are now calling upon it to perform is an obligation of this kind. If the medical profession would act together as one man for the protection of the morals and health of our public schools, is it for a moment to be supposed that it could not effectually and for ever suppress the base and degrading propaganda of a mere handful of filthy dealers in useless and dangerous drugs? The medical profession, we are convinced, is not at all aware of the esteem in which its leaders and itself as a whole are held by a thinking public. All the world is well convinced of two things concerning us: it knows that we are strenuous in our pursuit of new knowledge; it knows also that we are entirely honest and straightforward in the reports we give of our own practical advancement; it knows, moreover, by daily household experience, that the average doctor in his own speciality is eminently worthy of confidence and trust. Is it to be supposed then for a moment that the forty or fifty thousand medical men of these islands, if they could be brought up to the level of a great combined public demonstration in favour of any good cause whatever, could fail of an effect upon the intelligent public mind?

The medical profession, we are convinced, can effectually protect the health and morals of our thousands of public schoolboys if it will. What are the things to be aimed at in a crusade of this kind? The first thing to be done is to form an offensive and defensive alliance of medical men, teachers, and school managers; the next is to secure the sympathy and co-operation of the boys and their parents; and the third is to flood public opinion with the light of what we may call physiological morality, and to rouse it to a high pitch of moral and physiological enthusiasm. With the boys, the school managers, the parents, and the public thoroughly enlightened and on our side, a roused and enthusiastic medical profession could carry any reasonable measures through Parliament which its sober wisdom might consider to be necessary in the cause of school purity and health. It is for us, whose creed is physiological, whose aim is health and vigour for the State, whose trained eyes see clearly the blasting and ruinous effects upon youthful health and morals of filthy literature and filthier drugs, to shake ourselves free from our half-cynical lethargy and to insist that, at any rate in the name of medicine, the morals of boys shall not be polluted and the bodies of boys shall not be poisoned for the enrichment of conscienceless knaves.

The medical profession has never accustomed itself to act in its united capacity. What an infinite pity it is! There is not an organisation among us which can speak with a voice to which the whole public would listen with conviction. But there ought to be. The public knows less of us and of our science than of any other profession. It is, therefore, entirely dependent upon us for knowledge and guidance; and if we did but know how to guide it wisely it would gladly follow our lead. This question of public school degradation and public school robbery is only one of many in which, if we could be organised to act together with united and assured strength, we should be irresistible. A revolution must be brought about. The College of Physicians might take the initiative, but it will not; the British Medical Association might lead the way, but it cannot. Paralysis of concerted action is everywhere abroad. A revolution is imperative if medicine, as a body, is not to sink lower and lower towards the general paralysis of the incapable.

Thus far we have urged upon the medical profession to rouse itself for a great crusade against medical knavery in the interests of school morals and the general well-being. But though we have insisted that medical men are bound to take the lead in this crusade, we have not said that the public itself is absolved from every obligation in the matter. On the contrary, it is surely one of the very first of all the duties of the public to provide for its own protection. Should parents be indifferent to the threatened contamination of their boys at our public schools? Should school managers be indifferent? Should Parliament be indifferent? The Briton is slow to be roused in what he considers to be new causes. But is this a new cause? The health and purity of our school-boy life cannot be considered a new cause. What is new is the bold, insidious, and degrading temptation to vice which infamous and conscienceless scoundrels thrust upon ignorant youth. That a few public school-boys are willing and eager to be led into vice, and to be shown how to escape its dangers, no man of the world will deny. But that the majority hate vice, hate the temptations to it, hate those who for the base lust of gain promise them safe indulgence in it, every clean man is entirely assured. In a cause like this science may unite with morals, reason with religion, the man of the world with the man of the cloister and the laboratory. A clean youth and a clean manhood, a healthy body and a vigorous and uncontaminated mind are individual and imperial, present and life-ong advantages which cannot by any possibility be bought at too high a price.

